# Sociodemographic characteristics of SLE patients in a large metropolitan area with a high Afro-Caribbean population

**DOI:** 10.1186/ar3983

**Published:** 2012-09-27

**Authors:** J Cabas-Vargas, NM Patel, J Cohen, EM Ginzler

**Affiliations:** 1 State University of New York, Downstate Medical Center, Brooklyn, NY, USA

## Background

Systemic lupus erythematosus (SLE) is characterized by a wide spectrum of manifestations and severity, frequently affecting women and ethnic minorities. Health disparities among ethnic groups adversely impact medical care access, treatment choices and long-term outcomes. Over the past 30 years our lupus cohort has changed to include many patients of Afro-Caribbean ethnicity. Understanding ethnic and sociodemographic characteristics is critical to designing strategies to decrease health disparities and improve patient care.

## Methods

A demographic questionnaire was distributed to lupus patients from June 2009 to May 2012. All patients met ≥4 1982 ACR SLE criteria. Questions concerned date of diagnosis, place of birth, ethnicity, religion, main language, marital status, housing, education, employment, financial support, disability and medical insurance. Descriptive statistics were compared with similar demographic studies done in 1980 and 1994, including 164 and 169 patients respectively.

## Results

One hundred and fifty patients participated in the survey; 90% were women with average disease duration of 15 (0.4 to 52) years. Forty-seven percent were born in North America, 37% in the Caribbean and 15% in other regions, with a similar distribution in 1994 (47% North America and 45% Caribbean), but a notable change from 1980s demographics, with 74% born in North America and 22% in the Caribbean. The most common ethnicities were Black non-Hispanic (68%) followed by Black-Hispanic (17%) and White-Hispanic (5%). Currently the main spoken language is English (93.3%). Only 21.3% are married, with 54% never married. Eighty percent completed a high-school education, and 26.2% have full-time jobs while 51% are unemployed. The most common occupations are childcare, healthcare, and clerical. Average adjusted gross income is below New York State income ($39.438 per year vs. $59,519 per year). A total of 47.3% of patients receive disability with 40% reporting disability as the main source of financial support, an increase in disability status compared with 1980 (38.4%) and 1994 (46.1%). Access to healthcare and insurance plans increased compared with 1980 (53% Medicaid and 20.1% Medicare vs. 46% self-pay, 36% Medicaid and 20% private). Drug plan coverage is available for 85% of the current cohort.

## Conclusion

Although genetic variations highly influence disease patterns, adverse sociodemographic factors negatively influence disease course. Most of our patients come from Caribbean communities with limited education, high unemployment rate, low income and high disability rates. Despite improved access to healthcare, sociocultural barriers may limit access to optimal medical therapies, necessitating interventions to decrease health disparities. Studies comparing clinical characteristics and their influence on outcome of Afro-Caribbean populations and other ethnicities will help to improve medical care for this population.

